# Research on Low Temperature Performance of Emulsified Asphalt Cold Recycled Mixture and Improvement Measures Based on Fracture Energy

**DOI:** 10.3390/ma13143176

**Published:** 2020-07-16

**Authors:** Jialin Zhang, Mulian Zheng, Jianzhong Pei, Jiupeng Zhang, Rui Li

**Affiliations:** School of Highway, Chang’an University, Xi’an 710064, China; zjl2016@chd.edu.cn (J.Z.); zml@chd.edu.cn (M.Z.); zhjiupeng@chd.edu.cn (J.Z.); lirui@chd.edu.cn (R.L.)

**Keywords:** emulsified asphalt cold recycled mixture, SCB test, low temperature crack resistance, fracture energy

## Abstract

At present, there are no specific indicators and requirements for the low-temperature crack resistance of emulsified asphalt cold recycled mixture (CRME) in the Chinese road mixture specifications. In order to expand the application of this technology in the asphalt surface layer in cold areas, this paper studied the influence of 10 influencing factors on the low-temperature anti-cracking performance of CRME through the semicircular bending test (SCB) with fracture energy as the evaluation index. The research results show that the fracture energy index of the SCB test can be used to evaluate the low temperature crack resistance of CRME. After 10 kinds of influencing factors were analyzed, it was found that the biggest factor affecting the low-temperature cracking resistance of the mixture was the recycling agent, which had an effect on the fracture energy index of over 60%. Followed by cement, fiber and compaction work, the degree of influence exceeded 30%. Finally, combined with engineering application experience, some specific measures to improve the low-temperature anti-cracking performance of CRME were proposed.

## 1. Introduction

Emulsified asphalt cold recycling technology is one of the main ways of reclaimed asphalt pavement (RAP) in China’s asphalt pavement reconstruction and expansion projects. In some projects implemented early, the application level of emulsified asphalt cold recycled mixture (CRME) was relatively low, which was mainly used as a flexible upper or lower base. With the development of this technology, the performance of the mixture had been further improved, and its application level had gradually moved up. In some projects, it had been applied to the lower layers.

In terms of the evaluation of the anti-cracking performance of CRME, many scholars have conducted a lot of research in conjunction with the engineering application. Loria, McDaniel and Morian [1,2,3] investigated the use effect of multiple emulsified asphalt cold recycling sections implemented in the states of Nevada, Indiana and Bentley. The research results showed that the use of CRME could effectively reduce the number of cracks in the pavement. The antireflective cracking capacity of emulsified asphalt cold recycled pavement was two to three times that of the traditional direct overlay with hot-mix asphalt mixture, and the cost was only two-thirds of the hot-mix asphalt mixture. Charmot et al. evaluated the fracture energy of CRME using disk-shaped compact tension (DCT), and found that the peak value of CRME’s fracture strength was less than that of ordinary hot-mix asphalt. Compared with the hot asphalt mixture, it had a longer crack development path on the load-deformation fracture curve [4]. Gao et al. used the Arcan fracture test to evaluate the composite crack resistance of CRME. The research results showed that the crack development of CRME includes three stages: initiation, stable expansion and unstable expansion. During the fracture process, the development of the fracture always proceeds in the direction of the minimum energy consumption required to produce the fracture, mainly concentrated at the interface between the coarse aggregate and the emulsified asphalt mastic [5]. Yan et al. tested the stiffness modulus of CRME at different temperatures and stress levels [6]. In addition, Han et al. tested the flexural tensile strength and bending stiffness modulus of CRMEs with different latex content at different low temperatures. It was found that when the latex content was greater than 3%, CRME had better low temperature performance [7]. Ma et al. found that a reasonable content of RAP was conducive to improving the crack resistance of cold recycled mixtures. If the content of RAP was too large, the maximum bending strain of the mixture at low temperature would gradually decrease, and the cleavage strength would increase [8]. Some researchers have conducted more research on the material properties of the cement–CRME, and found that adding a small content of cement can effectively improve the initial strength of the mixture. As the content of cement increased, the bending and tensile properties, high temperature stability and water stability of CRME would increase. However, when the content of cement exceeded 2.0%, the improvement of the above properties was greatly reduced, and the low temperature performance and fatigue performance of the mixture were significantly reduced [9,10,11,12,13].

At present, the technical indicators for the emulsified asphalt plant-mix recycling in Chinese recycling technical specifications mainly include physical performance indicators (void ratio), strength indicators (indirect tensile strength (ITS) and stability) and water stability performance indicators (dry and wet ITS ratio, residual stability ratio and freeze–thaw ITS ratio) [14]. When the technology is applied to the lower layers of heavy traffic load grade highways, specific index requirements are proposed for the dynamic stability characterizing high-temperature performance. However, the current specification has not yet involved low-temperature performance indicators and standards. Therefore, it is necessary to study the low-temperature performance indicators and standards of the cold-mixed recycled mixture of the emulsified asphalt plant in order to promote the application of this technology in the asphalt surface layer in cold areas. The research achievements of scholars mostly focus on the effect of a single additive on the low-temperature crack resistance of the mixture, but there is no systematic study on the factors that affect the low-temperature crack resistance.

In this paper, through the fracture energy index of the semicircular bending test (SCB), the factors that affected the low-temperature crack resistance of CRME were evaluated, and specific performance improvement measures were proposed. 10 types of factors were studied, including recycling agent, SBR latex, fibers, different asphalt grade, compaction work, gradation, emulsified asphalt content, new crushed stone, cement and mineral powder.

## 2. Materials and Experiments

### 2.1. Materials

The Chinese recycling technical specification recommends the slow-setting cationic emulsified asphalt for CRME. In this paper, emulsified asphalt A, B and C were prepared by using three types of base asphalt with penetration of 50, 70 and 90 respectively. The test results are shown in Table 1. In Section 3.1.2 of this article, emulsified asphalt A, B and C, were used for comparative tests, and emulsified asphalt B was used in the remaining chapters.

Asphalt surface milling materials were provided by the Baomao Expressway Maintenance Project in Inner Mongolia. The RAP was divided into three grades: 0–4.75 mm, 4.75–9.5 mm and 9.5–31.5 mm. The strength grade of ordinary Portland cement was 32.5 MPa. The particle size of the new crushed stone was 9.5–19 mm.

### 2.2. Mixture Design

The material composition of CRME was: RAP1 (9.5–31.5 mm):RAP2 (4.75–9.5 mm):RAP3 (0–4.75 mm):new crushed stone (9.5–19 mm):mineral powder = 37:13:38:10:2. The synthetic gradation of CRME is shown in Table 2.

By establishing the dry density curve corresponding to different water content, the water content corresponding to the maximum dry density was the optimal water content. The optimal moisture content of the mixture was 4.0%. According to the air void, ITS and freeze–thaw ITS parameters of CRME, the optimal asphalt aggregate ratio was comprehensively determined.

In this paper, the basic formulation of the mixture was determined. On this basis, the influence of a single factor alone on the fracture energy of the mixture was studied. The basic formula of CRME was: emulsified asphalt B, the gradation 2, new crushed stone content (10%), emulsified asphalt content (3.5%), cement content (1.5%), mineral powder content (2%) and compaction work (150 + 75 times). No recycling agent, fiber and SBR latex were added.

### 2.3. Preparation of the Samples

#### 2.3.1. Big Marshall Sample Preparation Method

In this study, the large Marshall test samples were mainly used for the SCB test. According to the specification (JTG/T 5521-2019) Appendix F.7 [1], the second compaction method was used to form the large Marshall test samples.

**Figure 1:**
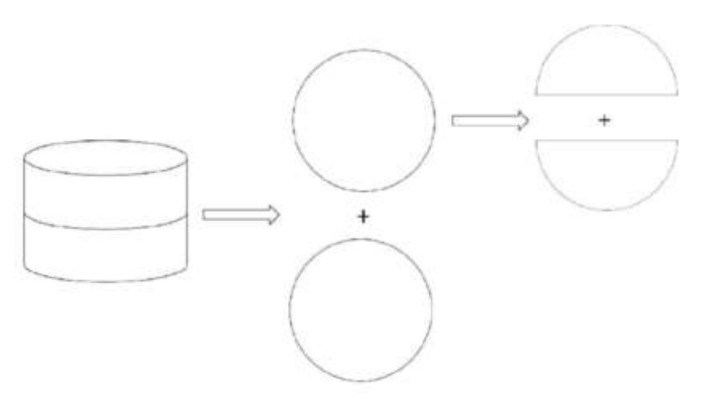
Sample preparation.

**Figure 2:**
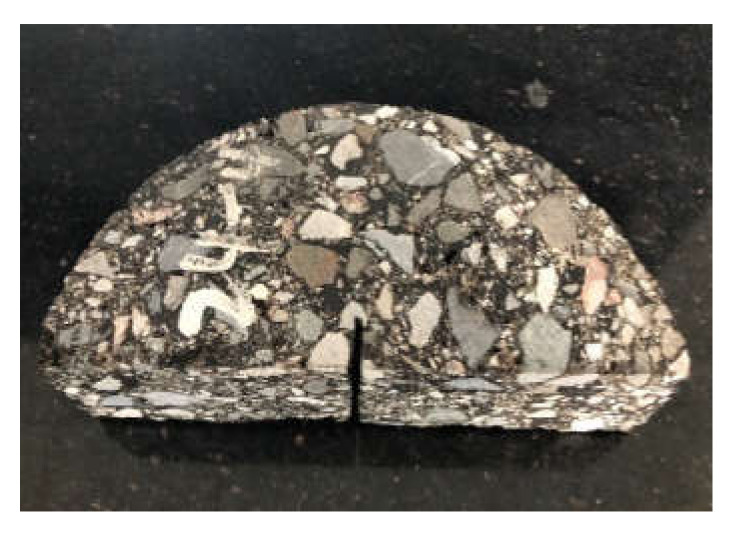
Semicircular bending sample.

(a) The mixed aggregate was added to the mixing machine, and water was added according to the calculated value and mixed. The mixing time was generally 60 s.
(b) Emulsified asphalt was then added, and the mixture was continuously mixed for 60 s.
(c) The uniformly mixed mixture was loaded into a large Marshall test mold (φ152.4 mm × 105 mm) and compacted. The samples were compacted 150 times on each side.
(d) The sample was placed sideways in a 60 °C blast oven together with the test mold to maintain a constant weight for 48 h.
(e) The sample and the test mold were taken out of the oven, and the second compaction was performed. The two sides of the sample were compacted 75 times each. Then the test samples was placed on the ground at room temperature to cool for at least 12 h and then demolded.
(f) The test samples was cut into four half cylinders. The size of the semi-circular sample was φ152.4 mm × 50 mm. Each semi-circular sample was cut at the midpoint of its diameter in a radial direction with a length of 15 ± 2.5 mm and a width of 2.5 ± 1 mm. Schematic diagram of sample cutting and finished samples are shown in Figure 1 and Figure 2.

#### 2.3.2. Standard Marshall Sample Forming Method

In this study, standard Marshall samples were used to detect the air void, ITS and freeze–thaw ITS of CRME. The size of the standard Marshall sample was φ101.6 mm × 63.5 mm. The difference between the forming methods of the two types of test samples was mainly the number of times of compaction, as shown in Table 3.

### 2.4. SCB Test

**Figure 3:**
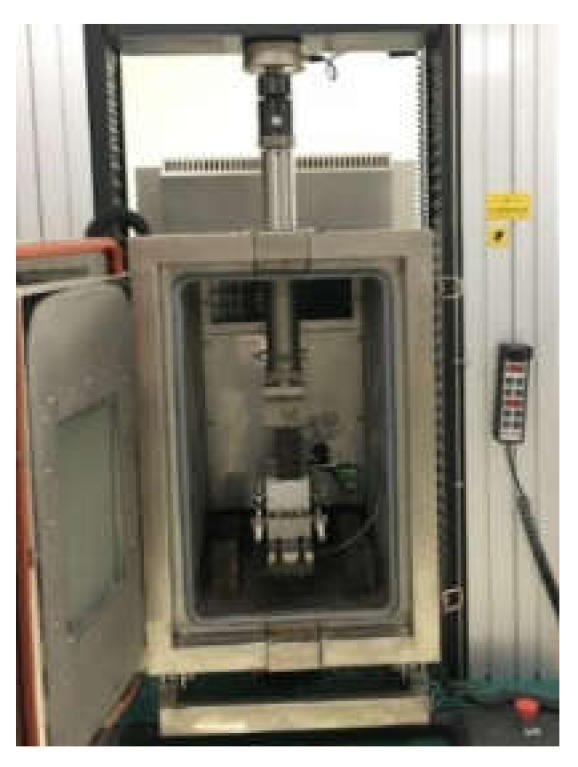
Semicircular bending test (SCB) test.

According to AASHTO SCB TP 105-13 (2015) and Chinese specification DB13/T 2020-2014, the universal material testing machine and incubator were used for testing. The samples were placed in an environmental incubator at −10 ± 0.5 °C for 4–6 h, and the loading rate was 1 mm/min. The distance between the fulcrums was 120 mm ± 0.5 mm. Eight parallel samples were tested for the same type of mixture. SCB test is shown in Figure 3.

The area A under the experimental curve was calculated according to Equation (1) [15].

(1) $$A=\overset{n}{\underset{i=0}{\sum }}\left[\left(x_{i+1}-x_{i}\right)y_{i}+0.5\left(x_{i+1}-x_{i}\right)\left(y_{i+1}-y_{i}\right)\right]$$

where:

*x_i_*—the vertical displacement of *i* step (mm);

*x_i+_*_1_—the vertical displacement of the *i +* 1 step (mm);

*y_i_*—applied load at the *i* step (kN);

*y_i+_*_1_—applied load at the *i +* 1 step (kN).

According to Equation (2) the fracture energy of the sample was calculated.

(2) $$G_{f}=10^{6}\frac{A}{b\times \left(h-a\right)}$$

where:

*G_f_*—fracture energy of test sample (J/m^2^);

*b*—test sample thickness (mm);

*h*—the radius of test sample (mm);

*a*—notch length of test sample (mm).

### 2.5. Indirect Tensile Strength (ITS) Test

**Figure 4:**
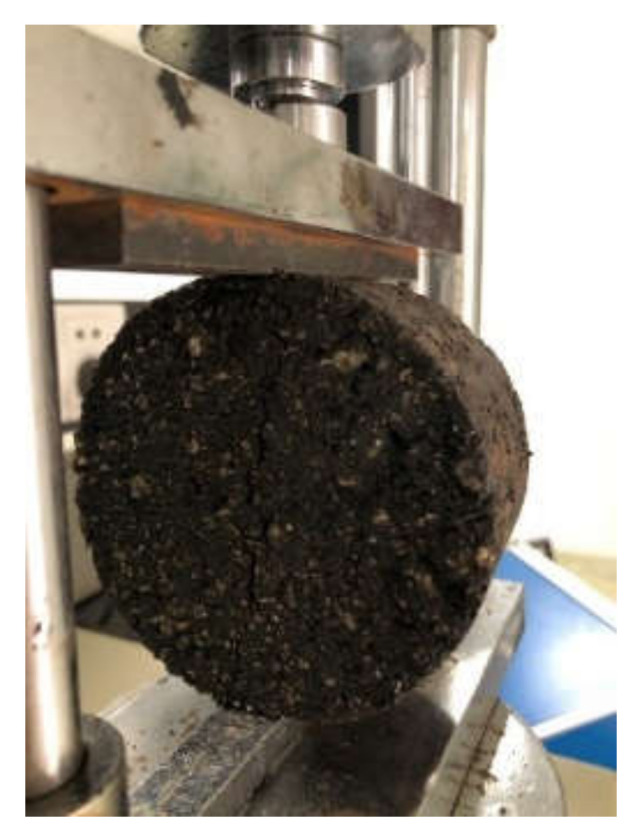
Indirect tensile strength (ITS) test.

The ITS of the mixture was tested according to the splitting test method (JTG E20-2011, T0716-2011) [2]. The samples were placed in a thermostatic water bath at 15 ± 0.5 °C and immersed in water for 2 h. Then, the test samples were removed from the sink and the surface was wiped clean. The test samples were placed on the loading table for testing, and the loading speed was 50 ± 5 mm/min. Four parallel samples were tested for the same type of mixture. ITS test is shown in Figure 4.

### 2.6. Freeze–Thaw ITS Test

According to Chinese specifications (JTG E20-2011, T0729-2011) [16], the freeze–thaw ITS test was performed to evaluate the water stability of CRME. The two groups of samples were formed according to the Marshall one-time compaction test and the Marshall two-times compactions test. The first set of samples were placed on a platform and stored at room temperature for use. The second group of samples were vacuum-saturated and maintained for 15 min, and then placed in water for 0.5 h under normal pressure. Subsequently, the samples were placed in a −18 °C for 16 h. Finally, the samples were then kept in a 60 °C water bath for 24 h. All the first and second groups of samples were immersed in a 25 °C water tank for not less than 2 h. The samples were taken out and the ITS of CRME were tested at a loading speed of 50 mm/min. The freeze–thaw ITS ratio was calculated. Four samples were tested for the same type of mixture.

### 2.7. Rutting Test

According to Chinese specifications (JTG E20-2011, T0719-2011), the high temperature performance of CRME was evaluated. A certain content of the mixture was put into a test mold (300 mm × 300 mm × 80 mm), which was firstly rolled in one direction for 2 round trips (4 times) and unloaded. The samples were turned in the direction, and then rolled 12 round trips (24 times) with the same load. The formed rutting samples were placed in a 60 °C blast oven for curing for 48 h. The rut test conditions were 60 °C and 0.7 MPa tire pressure. Three parallel samples were tested for the same type of mixture.

## 3. Results and Discussion

### 3.1. The Effect of Various Factors on the Low Temperature Performance of CRME

#### 3.1.1. Additive

(1) Recycling agent

The main components of asphalt are asphaltenes, gums, saturated components and aromatic components. Due to the long-term aging of the asphalt in the RAP material, the light components gradually decrease. The main component of the asphalt recycling agent is aromatic fragrance, which can supplement the light components in the asphalt and improve the ductility of the asphalt, thereby improving the low temperature crack resistance of the mixture [17].

**Figure 5:**
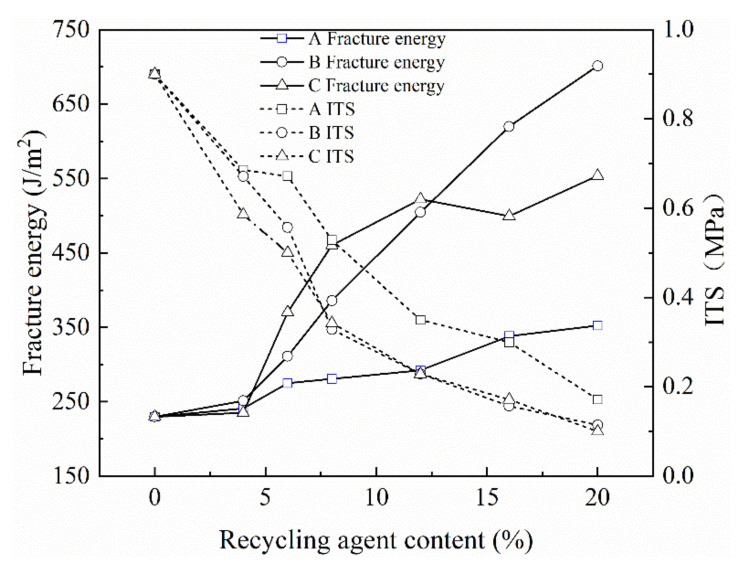
The effect of recycling agent on the fracture energy and ITS of the mixture.

In this study, three regenerators A, B and C were used in the RAP mixture. The addition ratio of recycling agent was 0%, 4%, 8%, 12%, 16% and 20% of the old asphalt content in the RAP material. The optimal content of emulsified asphalt after adding a recycling agent was 2.8%. The test results are shown in Figure 5.

It can be seen from Figure 5 that when the addition ratio of the recycling agent exceeds 4%, the three regenerators A, B and C can all improve the fracture energy of the mixture to a certain extent. The effect of recycling agents B and C on the fracture energy of the mixture is most obvious, and the effect of recycling agent A is relatively small. However, with the increase of the content of the recycling agent added, the ITS of the mixture showed a downward trend and the recycling agents B and C decreased the ITS more significantly.

According to the requirements of China’s recycled asphalt mixture specifications, when CRME was applied to heavy traffic grade highways, its ITS should be greater than 0.6 MPa. The ITS of 0.6 MPa was taken as the boundary condition, it can be seen from Figure 5 that when the addition contents of the recycling agents A, B and C were 10%, 7% and 6.5%, respectively. The fracture energy increased by 23.9%, 52.3% and 74.1%, which also shows that the recycling agent C had the most obvious increase in the fracture energy of the mixture. It was also recommended that the content of recycling agent C should be 5–7%.

(2) SBR latex

Styrene-butadiene rubber (SBR) latex was a stable emulsion made from butadiene and styrene polymerized at low temperature. Related studies had shown that the lipophilic groups of SBR were compatible with some linear paraffins in asphalt, which could improve the low temperature performance of the mixture [18]. The SBR used was imported styrene-butadiene latex with a solid content of 65% and a density of 0.95 ± 0.02 g/cm^3^.

**Figure 6:**
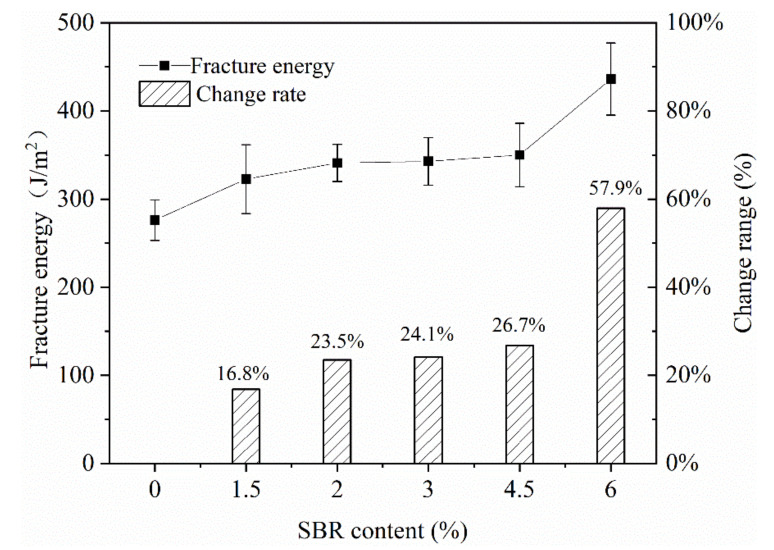
The effect of SBR latex on the fracture energy of the mixture.

In this study, the different contents of SBR latex were used to improve the low temperature crack resistance of the mixture. SBR latex was added by 0 wt%, 1.5 wt%, 2 wt%, 3 wt%, 4.5 wt% and 6 wt% of the emulsified asphalt. It can be seen from Figure 6 that as the content of SBR latex increased, the fracture energy of CRME gradually increased. However, when the content of SBR latex added was between 1.5% and 4.5%, the fracture energy of the mixture increased by 16.8% to 26.7%, and the change range was limited. When the content of SBR added reached 6%, the increase in fracture energy was larger, reaching 57.9%. In practical engineering applications, considering the high price of SBR latex, the high content of SBR would greatly increase the material cost of a cold recycled mixture. Therefore, it was recommended that the content of SBR latex was 2–3%.

(3) Fiber

The addition of fiber in the asphalt mixture can form network reinforcement and improve the crack resistance of the mixture [19,20]. In this paper, two types of basalt fibers with lengths of 6 mm and 12 mm were used. The contents of fibers added were 0%, 0.2%, 0.3% and 0.4% of the weight of the mixture.

**Figure 7:**
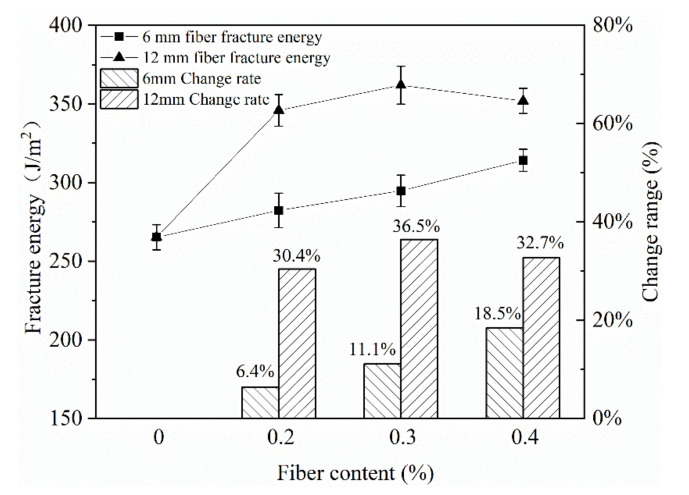
The influence of fiber on fracture energy.

It can be seen from Figure 7 that as the content of basalt fiber increased, the fracture energy of the mixture shows a gradual increasing trend. This was mainly because the uniformly dispersed fibers bridged each other, forming a space network support system in CRME. On the one hand, the fibers could play a kind of “reinforcement” effect on the emulsified asphalt mastic and aggregates, limiting the displacement of the aggregates and restricting the failure of the emulsified asphalt mastic. On the other hand, after microcracks were generated, the fibers isolated the damaged area. The fibers across the two ends of the crack constrained the deformation of the crack, hindered the continued development of the crack and served as an isolation function. This could greatly reduce stress concentration, delay or even prevent the occurrence and development of damage cracks. The fracture energy of the mixture added with 12 mm fiber was higher than that of the mixture added with 6 mm fiber, and the crack resistance effect was better.

**Figure 8:**
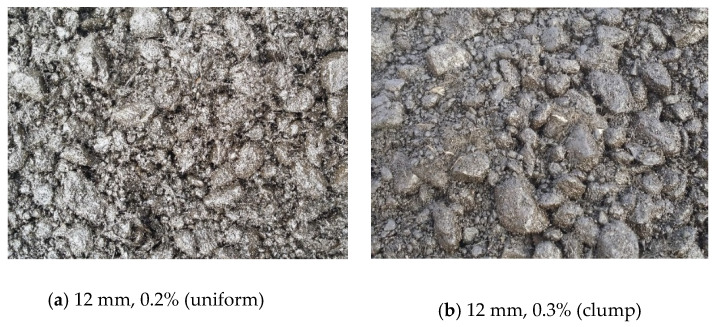
Fiber mixture.

It can be seen from Figure 8 of the mixing effect of the mixture that the mixture with the addition of 6 mm fibers was more uniformly mixed. When the content of 12 mm fiber added increased from 0.2% to 0.3%, the mixture began to appear uniformly dispersed and obviously agglomerated from being relatively uniform. Therefore, it was recommended to use 12 mm basalt fiber to improve the low temperature crack resistance of the mixture, and the addition content should be 0.2%.

#### 3.1.2. Mixture Material Formula

(1) Asphalt type

In China, three types of road asphalts with penetration levels of 50 #, 70 # and 90 # were mainly used. In the hot areas, 50 # or 70 # asphalt was mostly used, and 90 # bitumen was more used in the cold northern areas such as Inner Mongolia and Xinjiang. In this study, three types of base asphalt were used to prepare emulsified asphalts A, B and C.

**Figure 9:**
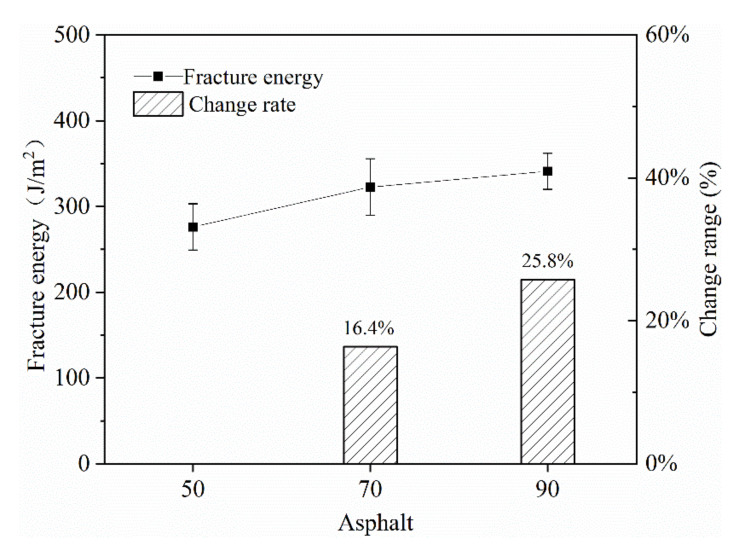
The effect of different grades of asphalt on the fracture energy of the mixture.

From Figure 9, it is found that with the increase of the base asphalt number, the fracture energy of the mixture was gradually increasing. Based on the fracture energy of the mixture prepared with 50 # asphalt, the mixture with 90 # asphalt had the best fracture energy, and the increase in fracture energy was 25.8%. The fracture energy of the mixture using 70 # asphalt was next, with an increase of 16.4%. It shows that the higher the asphalt number, the better the ductility of asphalt, the better the low temperature crack resistance of the mixture.

(2) Gradation

**Figure 10:**
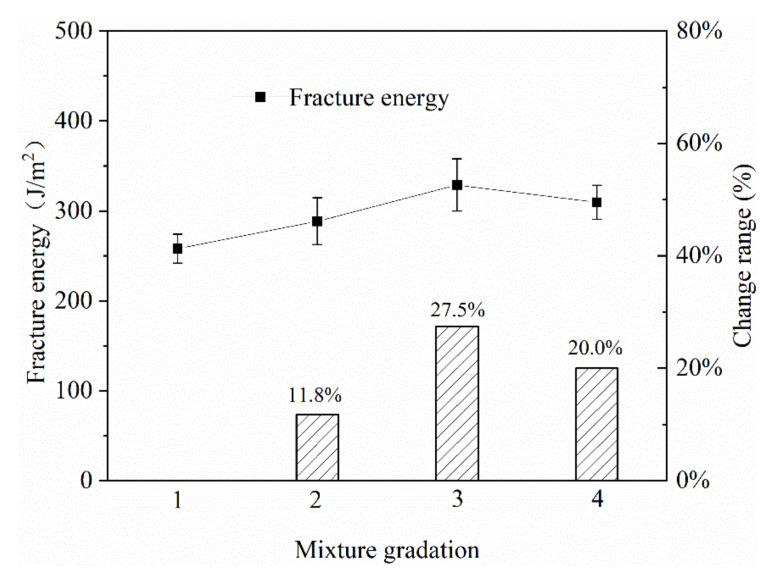
The effect of gradation on fracture energy.

The research results showed that gradation had a significant effect on the mechanical strength and road performance of asphalt mixtures [21,22]. Based on engineering experience, four gradations were selected, as shown in Table 4. Among them, the passing rate of the 4.75 mm key screen holes of Gradation 1, 2, 3 and 4 were 32.2%, 35.7%, 40% and 45.2% respectively. The results of the SCB tests for the four gradations were shown in Figure 10.

It can be seen from Figure 10 that in gradation 3, when the pass rate of 4.75 mm was 40%, an inflection point appeared in the fracture energy curve. When the pass rate of 4.75 mm increased from 32.2% to 40%, the fracture energy of the mixture gradually increased. When the pass rate of 4.75 mm reached 45.2%, the fracture energy of the mixture showed a downward trend. Compared with the gradation with a pass rate of 4.75 mm of 32.2%, the rupture energy was increased by 27.5% when the pass rate of 4.75 mm was 40%, indicating that the thickness of the mixture gradation had a significant effect on the fracture energy of the mixture.

(3) Emulsified asphalt content

**Figure 11:**
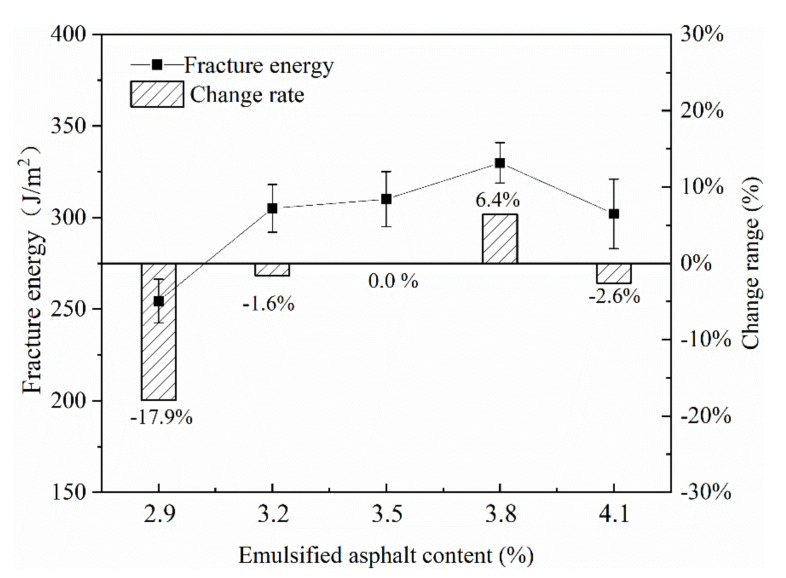
Effect of emulsified asphalt content on fracture energy.

It can be seen from Figure 11 that as the content of emulsified asphalt continued to increase, the fracture energy of the mixture showed an increasing trend. When the content of emulsified asphalt exceeded 3.8%, the fracture energy decreased. When designing the mix ratio of the mixture this time, the optimal content of emulsified asphalt was 3.5%, indicating that the appropriate increase in the content of emulsified asphalt was 0.3%, that is, when it reached 3.8%, the fracture energy of the mixture was slightly increased by 6.4%. However, when the content of emulsified asphalt was reduced by 0.6–2.9% from the optimal emulsified asphalt content of 3.5%, the mixture’s fracture energy decreased by 17.9%. It shows that the content of emulsified asphalt could be appropriately increased by 0.3% in the design of the mix proportion. However, the adjustment range should not be too large, especially when the content of emulsified asphalt was too low, it would have a greater impact on the fracture energy.

(4) Crushed stone content

When the cold milling process was used on the asphalt pavement, large-grain crushed stone usually had different degrees of crushing. It was usually necessary to add some new coarse aggregates to adjust the synthetic gradation of the mixture. The new coarse aggregate can improve the ITS and rutting stability of the mixture.

**Figure 12:**
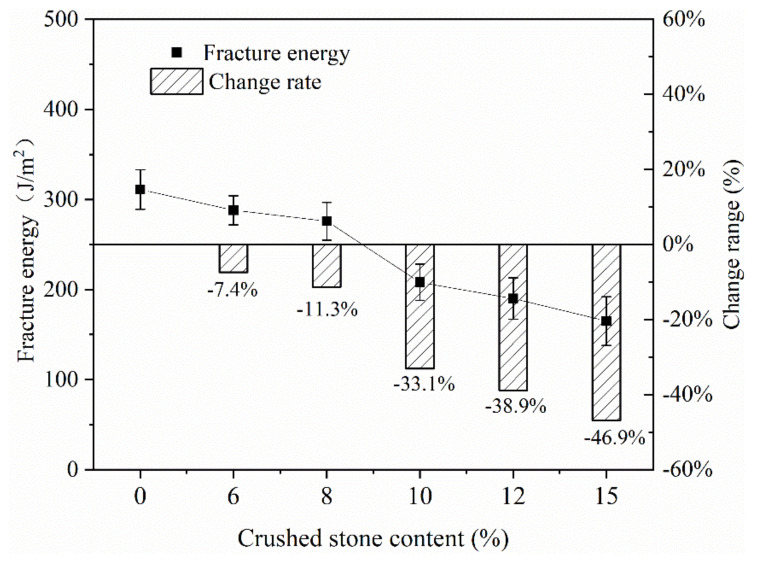
Effect of crushed stone content on fracture energy.

In order to compare the effects of different crushed stone additions on the fracture energy of the mixture, the new crushed stone additions were adjusted to 0%, 6%, 8%, 10%, 12% and 15%, respectively. The test results are shown in Figure 12. From the test results, it can be seen that as the content of crushed stone increased, the fracture energy of the mixture gradually decreased. The fracture energy without the addition of new crushed stone mixture was the largest, 311 J/m^2^. When the content of crushed stone increased from 6% to 15%, the fracture energy of the mixture decreased by 7.4–46.9%. Especially when the content of crushed stone was 15%, the fracture energy was only 165 J/m^2^ and the drop rate was nearly half. It showed that the addition of new crushed stone in the mixture had a significant adverse effect on its low temperature crack resistance.

(5) Cement content

The research results show that after adding a small content of cement to CRME, the hydration reaction of the cement and the emulsified asphalt demulsification to form the asphalt film adhesion RAP material simultaneously. The hydrate and the bitumen membrane were intertwined with each other independently and permeably, forming a spatial three-dimensional network structure wrapped around the RAP material. This spatial three-dimensional network structure not only ensured that the mixture had a certain strength, but also prevented excessive deformation of the mixture when the asphalt was softened under high temperature conditions. Therefore, in actual engineering applications, a small content of cement was generally added to improve the water stability, early strength and rutting resistance of CRME [21,23,24].

**Figure 13:**
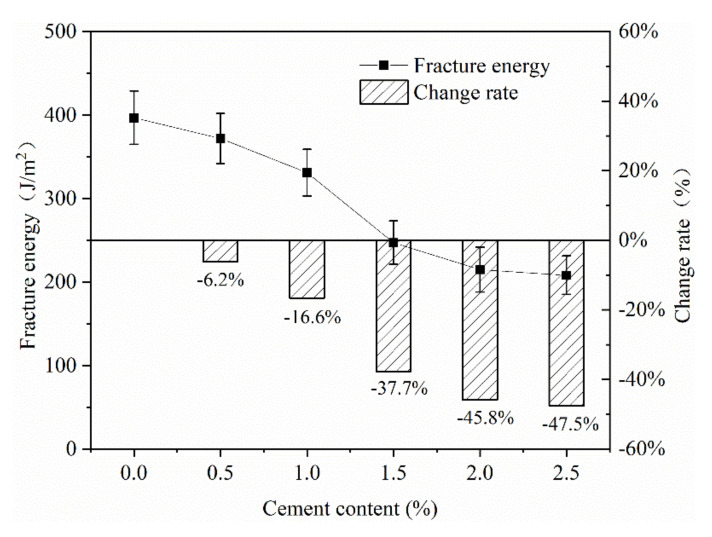
Effect of cement content on fracture energy.

In this paper, different contents of cement were added to the mixture. As can be seen from Figure 13, with the addition of cement contents, the fracture energy of the mixture gradually decreased, and the range of change was relatively large. Without adding cement, the fracture energy of the mixture was 397 J/m^2^. When the content of cement added reached 2.5%, the fracture energy dropped to 208 J/m^2^. The difference in cement contents was 2.5%, and the difference in fracture energy was almost double, which shows that adding cement to the mixture was not conducive to the low-temperature crack resistance of the mixture, and the effect was very obvious.

(6) Mineral powder content

**Figure 14:**
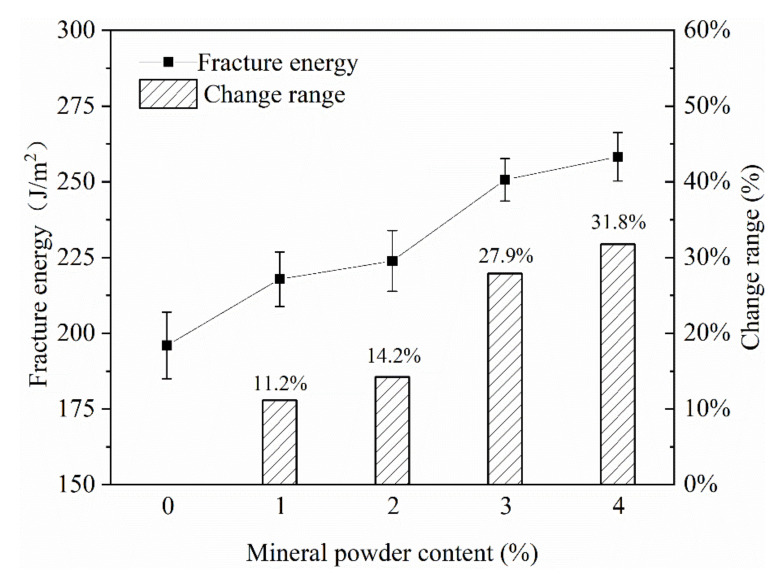
The effect of mineral powder content on fracture energy.

The addition of mineral powder in the emulsified asphalt mixture can strengthen the coating ability of the asphalt on the mineral material, improve the strength of the emulsified asphalt mastic and increase the strength of the mixture. In this paper, different contents of mineral powder were added to the mixture. It can be seen from Figure 14 that as the content of mineral powder increased, the fracture energy of the mixture continued to increase. Especially when the addition content of mineral powder reached 3–4%, the fracture energy of the emulsified asphalt mixture increased by 27.9–31.8%, indicating that adding mineral powder to the mixture can help improve the low-temperature crack resistance of the mixture [25,26].

During the test, it was also found that after the addition of mineral powder reached 4%, the test sample of the mixture showed a dark yellow color. Moreover, after the first compaction of some test samples, when the test mold was placed on the ground, the test samples will automatically fall off from the test mold. Conducive to the strength of the mixture. Therefore, the recommended content of mineral powder should be 2–3%.

#### 3.1.3. Compaction Work

Compaction is an important process of asphalt pavement construction. The quality of compaction directly affects the strength and stability of asphalt pavement and is related to the road performance of asphalt pavement [27]. An important reason for the serious early damage of some highway asphalt pavements is the poor compaction effect. This results in a large pavement void and low strength, which can cause water damage and rutting on the asphalt pavement.

In this paper, different compaction times were used to simulate different compaction effects on the construction site, and the low temperature crack resistance of the mixture was tested. The first compaction times of the Big Marshall sample were 75, 100, 125, 150, 175 and 200 times, respectively, and the second compaction times were 75 times.

**Figure 15:**
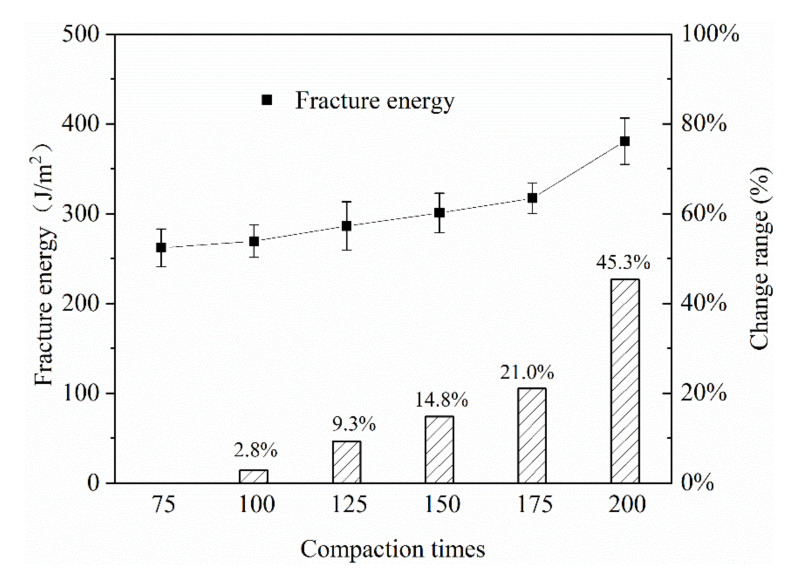
The impact of compaction times on the fracture energy of the mixture.

It can be found from Figure 15 that as the number of mixture compactions increased, the fracture energy shows a gradually increasing trend. Compared with the first compaction times of 75 times, when the compaction work reached 200 times, the fracture energy of the mixture was increased by 45.3%, and the improvement effect was very significant.

The method of forming the large Marshall test samples in the current recycling technical specifications in China was 75 times for the first compaction, and 37 times for the second compaction after curing at 60 °C for 48 h. The compaction work of the molding method in the current specifications needed to be considered for further improvement.

### 3.2. Research on Low Temperature Performance Improvement Measures

#### 3.2.1. Analysis of Influencing Factors

**Figure 16:**
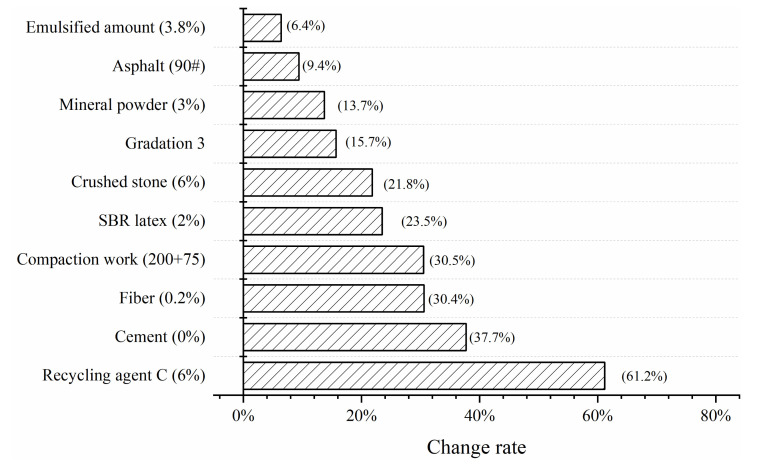
The sorting diagram of influencing factors of fracture energy.

The standard formula of emulsified asphalt cold recycled mixture in this paper was: emulsified asphalt (B), gradation (2), new crushed stone (10%), emulsified asphalt content (3.5%), cement (1.5%), mineral powder content (2%) and compaction work (150 + 75 times). No recycling agent, fiber and SBR latex were added. According to the above research results of the mixture formula, additives and compaction work, the results are shown in Table 5 and Figure 16.

It can be seen from Figure 16 that the biggest factor affecting the fracture energy of CRME was the recycling agent, which affected more than 70%. Followed by cement, fiber, compaction work and the degree of influence was between 30% and 40%. SBR latex, crushed stone, gradation and mineral powder were arranged in the back, and the degree of influence was between 10% and 25%. Asphalt designation and emulsified asphalt content had the smallest impact on fracture energy, with an impact of less than 10%.

#### 3.2.2. Performance Comparison of Different Measures

Based on the above research results, the influence of various factors on CRME fracture energy was fully considered, and combined with engineering application experience, four schemes were formulated. Among them, scheme B was the basic formula in this paper to study the influence of a single factor. The test schemes were shown in Table 6. Scheme A is a formula formulated according to the compaction molding method required by the current specifications (75 times for the first compaction, 48 h curing at 60 °C, and 37 times for the second compaction).

CRME was a normal temperature mixture, which is more difficult to compact than hot mixed asphalt mixture [28,29,30,31]. Therefore, the compaction work was usually increased to improve the compactness of CRME. Scheme B is a formula developed after adding compaction work (150 times for the first compaction, 48 h of curing at 60 °C and 75 times for the second compaction), and the remaining factors are the same as those in Scheme A.

During the construction of the asphalt pavement major repair project, it was necessary to shorten the curing time of the recycled mixture as much as possible, and open the traffic as soon as possible to reduce the interference of the construction project on the traffic. Considering various factors to improve the fracture energy of the recycled mixture, scheme C was proposed. Secondly, due to the influence of traffic channelization, the lane immediately after the construction may face the consequences of a sharp increase in traffic. It was usually necessary to improve the rutting resistance of the recycled mixture. Adding a small content of cement to the mixture could improve the early strength, water stability and resistance to rut deformation of the mixture [32,33,34]. Therefore, in order to improve the low-temperature fracture energy performance of the mixture and meet the characteristics of engineering needs, 1% cement and 6% crushed stone were added in scheme C.

In addition, considering that cement has a greater impact on the fracture energy of the mixture, scheme D does not use cement on the basis of scheme C. The rest of the factors are the same as scheme C.

**Figure 17:**
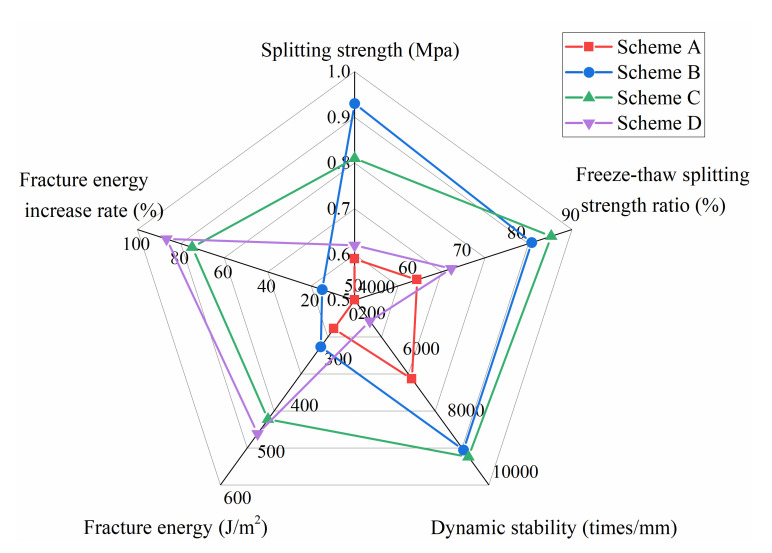
Comparison of mixture performance.

According to the above four formulas, a complete set of tests were conducted on the mixture void ratio, ITS, freeze–thaw ITS ratio, dynamic stability and fracture energy. The test results are shown in Table 7 and Figure 17. It should be noted that only the large Marshall test samples was used in the SCB test, and the rest of the mixture test used the standard Marshall test.

According to the Chinese recycling technical specification, CRME used on heavy traffic grade highways must have an ITS greater than 0.6 MPa and a freeze–thaw ITS greater than 75%. It can be seen from Table 7 that scheme A had a high air void due to insufficient compaction work. The ratio of ITS to freeze–thaw ITS did not meet the specification requirements.

On the basis of scheme A, the compaction work of scheme B had doubled. From the test results, it can be seen that the performance of the mixture was significantly better than scheme A, and the low temperature fracture energy index was increased by 14.9%. It showed that the increase of compaction work had a great influence on the performance of the mixture. Compared with scheme A and scheme B, the fracture energy of scheme C increased by 74.8% and 52.2%, respectively. The remaining performance was basically the same as scheme B. Among them, the air void, water stability performance and high temperature performance index were slightly higher than that of scheme B. However, due to the influence of the recycling agent, its ITS value was slightly lower. Compared with scheme C, scheme D (without cement) had a smaller increase in fracture energy, but the water stability performance and high temperature performance decreased significantly. Based on the above factors, scheme C was recommended to improve the performance of various materials of the mixture, especially the low temperature performance.

## 4. Conclusions

In this paper, through the fracture energy index of the SCB test, the effect of 10 influencing factors on the low-temperature crack resistance of CRME was studied. The following conclusions can be drawn.

(1) The effects of 10 factors, such as a recycling agent, SBR latex, fiber and compaction work, on the fracture energy of the mixture were studied, and the influencing factors were ranked. The biggest factor was the recycling agent, which affected the fracture energy index by more than 60%, followed by fiber and compaction work, and the influence degree exceeded 30%. In addition, the addition of cement and new crushed stone in CRME reduced the low temperature performance of the mixture.

(2) Based on the experimental results and previous engineering experience, Scheme C was proposed as an engineering formulation to improve the low temperature crack resistance of CRME in this paper. In addition, the passing rate of the 4.75 mm sieve should be about 40%. Penetration 90 asphalt was recommended to prepare emulsified asphalt, and the optimal content of emulsified asphalt was 2.8%. The compaction work of the large Marshall sample was adjusted from 150 times to 200 times in the first compaction, and the second compaction was still 50 times. The test results showed that the improved formula could increase the fracture energy by more than 70%.

## Figures and Tables

**Table 1:** Technical requirements and test results of emulsified asphalt.

Test Items		Unit	Technical Requirements	Emulsified Asphalt A	Emulsified Asphalt B	Emulsified Asphalt C
Breaking speed		/	Slow-setting	Slow-setting	Slow-setting	Slow-setting
Particle charge		/	Cationic (+)	Cationic (+)	Cationic (+)	Cationic (+)
Residual content on sieve (1.18 mm sieve)		%	≤0.1	0.02	0.01	0.01
Viscosity	Engra Viscometer E25	-	2–30	5.35	6.51	6.19
Evaporation residue	Residual content	%	≥62	62.3	62.5	62.7
	Solubility	%	≥97.5	99.9	99.5	99.8
	Penetration (25 °C)	0.1 mm	50–150	56.7	65.1	78.5
	Ductility (15 °C)	cm	≥40	92.1	≥100	≥100
Adhesion to coarse aggregates, covering area		/	≥2/3	≥2/3	≥2/3	≥2/3
Mixing test with coarse and fine aggregate		/	Uniform	Uniform	Uniform	Uniform
Storage stability	1 d	%	≤1	0.3	0.3	0.1
	5 d	%	≤1	0.3	0.3	0.1

**Table 2:** Synthetic gradation of emulsified asphalt cold recycled mixture (CRME).

Sieve (mm)	The Passing Rate (%)												
	31.5	26.5	19	16	13.2	9.5	4.75	2.36	1.18	0.6	0.3	0.2	0.1
Synthetic gradation	100	100	93.1	82.3	70.6	57.1	35.7	22.4	14.7	8.4	4.7	3.3	2.4
Upper limit	100	100	/	/	80	/	60	45	/	/	20	/	7
Lower limit	100	80	/	/	60	/	25	15	/	/	3	/	1

**Table 3:** Correspondence of compaction times of two types of Marshall samples.

Sample Type	Number of the First Compactions			Number of the Second Compactions	
Standard Marshall test samples	50	100	133	25	50
Big Marshall Test Samples	75	150	200	37	75

**Table 4:** Four types of mixture gradation.

Sieve Size (mm)		31.5	26.5	19	16	13.2	9.5	4.75	2.36	1.18	0.6	0.3	0.15	0.075
Gradation 1		100	100	92.4	80.6	67.8	53.3	32.2	20.3	13.4	7.7	4.4	3.2	2.3
Gradation 2		100	100	93.1	82.3	70.6	57.1	35.7	22.4	14.7	8.4	4.7	3.3	2.4
Gradation 3		100	100	93.3	82.8	71.3	58.5	40	25.0	16.3	9.2	5	3.4	2.4
Gradation 4		100	100	93.8	84.3	74	62.5	45.2	28.1	18.3	10.1	5.4	3.6	2.5
Gradation	Upper limit	100	100	/	/	80	/	60	45	/	/	20	/	7
	Lower limit	100	80	/	/	60	/	25	15	/	/	3	/	1

**Table 5:** Influence of single factor change on fracture energy.

Influencing Factors	The Basic Formula	The Change of Single Factor	The Increase of Fracture Energy
Emulsified amount	0	0.6%	61.2%
Cement	1.5%	0%	37.7%
Mineral powder	0	0.2%	30.4%
Gradation	150 + 75	200 + 75	30.5%
Crushed stone	0	2.0%	23.5%
SBR latex	10.0%	8.0%	21.8%
Compaction work	35.70%	40.0%	15.7%
Fiber	2.0%	3.0%	13.7%
Asphalt type	70 #	90 #	9.4%
Recycling agent C	3.5%	3.8%	6.4%

**Table 6:** Four types of mixture formula.

Name	Scheme A	Scheme B	Scheme C	Scheme D
Recycling agent	0	0	6.0%	6.0%
fiber	0	0	0.2%	0.2%
Compaction times (Big Marshall sample)	75 + 37	150 + 75	200 + 75	200 + 75
Compaction times (Standard Marshall sample)	50 + 25	100 + 50	133 + 50	133 + 50
SBR latex	0	0	2.0%	2.0%
gravel	10.0%	10.0%	6.0%	6.0%
cement	1.5%	1.5%	1.0%	0.0%
Grading (4.75 mm pass rate)	35.70%	35.70%	40.0%	40.0%
Mineral powder	2.0%	2.0%	3.0%	3.0%
Asphalt penetration	70	70	90	90
The best content of emulsified asphalt	3.5%	3.5%	2.8%	2.8%

**Table 7:** Comparison of mixture performance.

Index	Scheme A	Scheme B	Scheme C	Scheme D
Void ratio (%)	11.6	9.8	8.2	8.5
ITS (MPa)	0.58	0.93	0.81	0.62
Freeze–thaw ITS ratio (%)	61.5	82.6	86.2	67.8
Dynamic stability (times/mm)	5562	8865	9090	4681
Fracture energy (J/m^2^)	262	301	458	489
Fracture energy increase rate (%)	/	An increase of 14.9% compared to option A	Compared with schemes A and B, an increase of 74.8% and 52.2% respectively	Compared with schemes A and C, an increase of 86.6% and 6.8% respectively
